# Supplementary material for: Temporally resolved and interpretable machine learning model of GPCR conformational transition
Source: Nat Commun. 2025 Dec 6;17:257. doi: 10.1038/s41467-025-66958-4 (PMC12783649; doi:10.1038/s41467-025-66958-4)
Supplement: Supplementary file 1 — Supplementary Information [file 41467_2025_66958_MOESM1_ESM.pdf]

Supplementary Information

Temporally Resolved and Interpretable Machine Learning Model of GPCR conformational transition

Babgen Manookian[1], Elizaveta Mukhaleva[1,2], Grigoriy Gogoshin[1], Supriyo Bhattacharya[1], Sivaraj Sivaramakrishnan[3], Nagarajan Vaidehi[1,2], Andrei S. Rodin[1,2], Sergio Branciamore[1,2,*]

[1] *Department of Computational and Quantitative Medicine, Beckman Research Institute of the City of Hope, Duarte, CA*

[2] *Irell and Manella Graduate School of Biological Sciences, Beckman Research Institute of the City of Hope, Duarte, CA*

[3]*Department of Genetics, Cell and Developmental Biology, University of Minnesota, Minneapolis, MN*

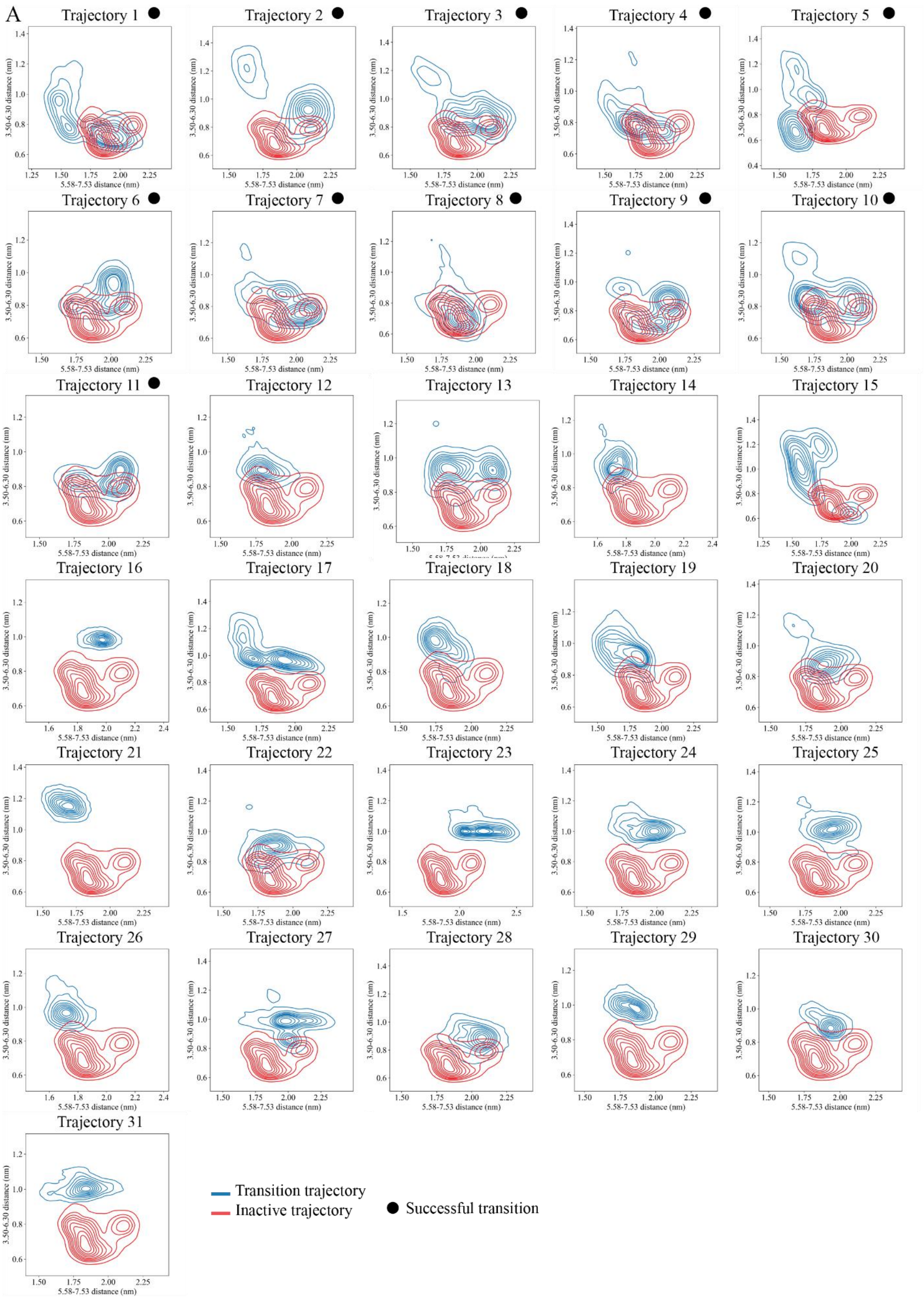
A
Trajectory 1
Trajectory 2
Trajectory 3
Trajectory 4
Trajectory 5
Trajectory 6
Trajectory 7
Trajectory 8
Trajectory 9
Trajectory 10
Trajectory 11
Trajectory 12
Trajectory 13
Trajectory 14
Trajectory 15
Trajectory 16
Trajectory 17
Trajectory 18
Trajectory 19
Trajectory 20
Trajectory 21
Trajectory 22
Trajectory 23
Trajectory 24
Trajectory 25
Trajectory 26
Trajectory 27
Trajectory 28
Trajectory 29
Trajectory 30
Trajectory 31
3.50-6.30 distance (nm)
5.58-7.53 distance (nm)
Transition trajectory
Inactive trajectory
Successful transition

Supplementary Fig. 1. (A) Density distribution of inter-residue distances between TM5-TM7 and TM3-TM6 from 31 transition trajectories and inactive state trajectories of D_2R. Kernel density estimation plots show the relationship between the TM5-TM7 (5.58-7.53) and TM3-TM6 (3.50-6.34) distances measured in nm. The blue contours represent the density distribution of transition trajectories, while the red contours correspond to the inactive trajectory. Black dot - successful transition trajectory.

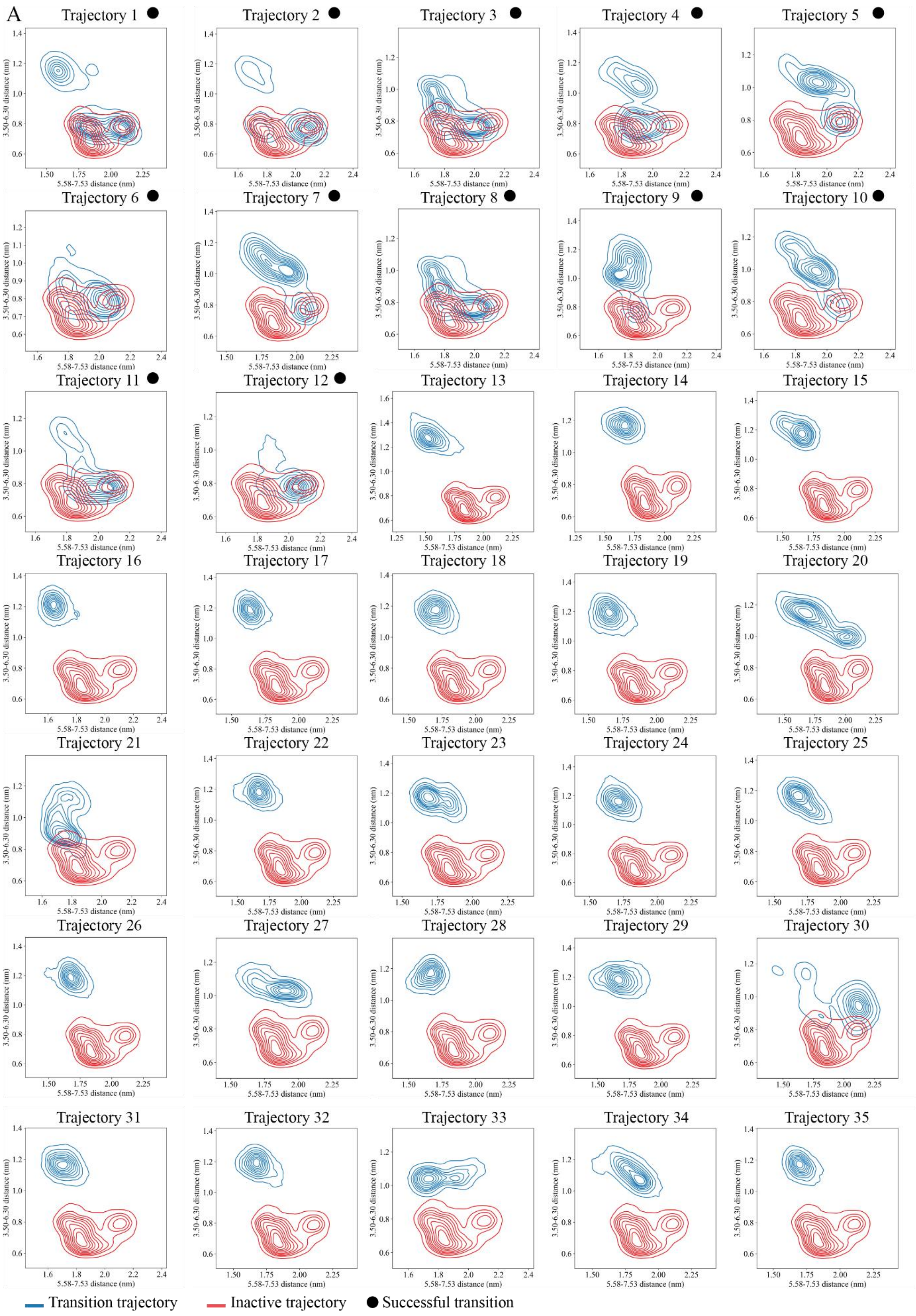
A
Trajectory 1
Trajectory 2
Trajectory 3
Trajectory 4
Trajectory 5
Trajectory 6
Trajectory 7
Trajectory 8
Trajectory 9
Trajectory 10
Trajectory 11
Trajectory 12
Trajectory 13
Trajectory 14
Trajectory 15
Trajectory 16
Trajectory 17
Trajectory 18
Trajectory 19
Trajectory 20
Trajectory 21
Trajectory 22
Trajectory 23
Trajectory 24
Trajectory 25
Trajectory 26
Trajectory 27
Trajectory 28
Trajectory 29
Trajectory 30
Trajectory 31
Trajectory 32
Trajectory 33
Trajectory 34
Trajectory 35
3.50-6.30 distance (nm)
5.58-7.53 distance (nm)
Transition trajectory
Inactive trajectory
Successful transition

Supplementary Fig. 2. (A) Density distribution of inter-residue distances between TM5-TM7 and TM3-TM6 from 31 transition trajectories and inactive state trajectories of D_3DR. Kernel density estimation plots show the relationship between the TM5-TM7 (5.58-7.53) and TM3-TM6 (3.50-6.34) distances measured in nm. The blue contours represent the density distribution of transition trajectories, while the red contours correspond to the inactive trajectory. Black dot - successful transition trajectory.

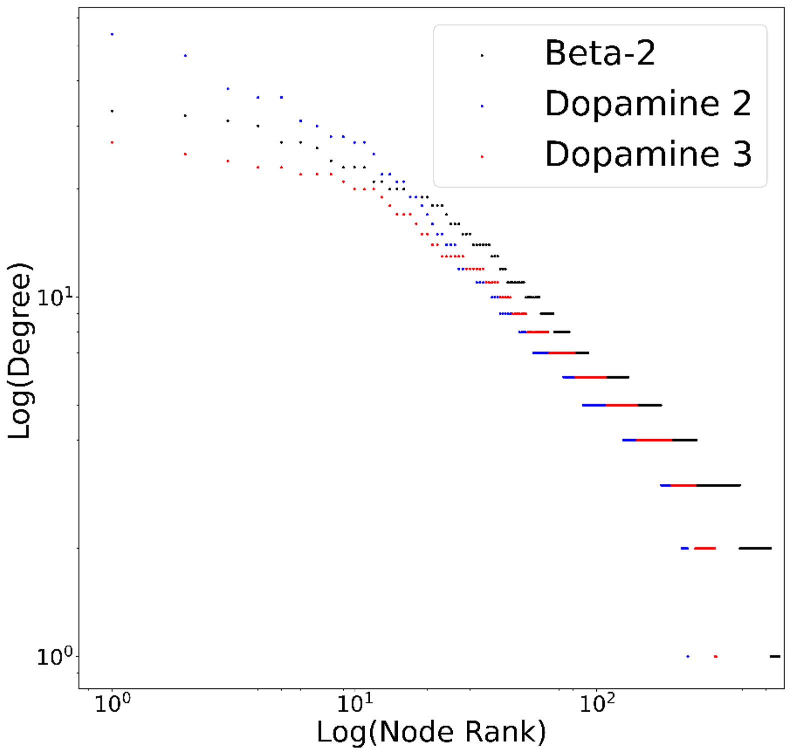

Supplementary Fig. 3. Degree distribution for all GPCR systems in the study. The universal graph constructed for each system was used to compute the distribution of degrees for nodes. The log-log plot highlights a near power-law distribution where a small number of nodes have the most connections.

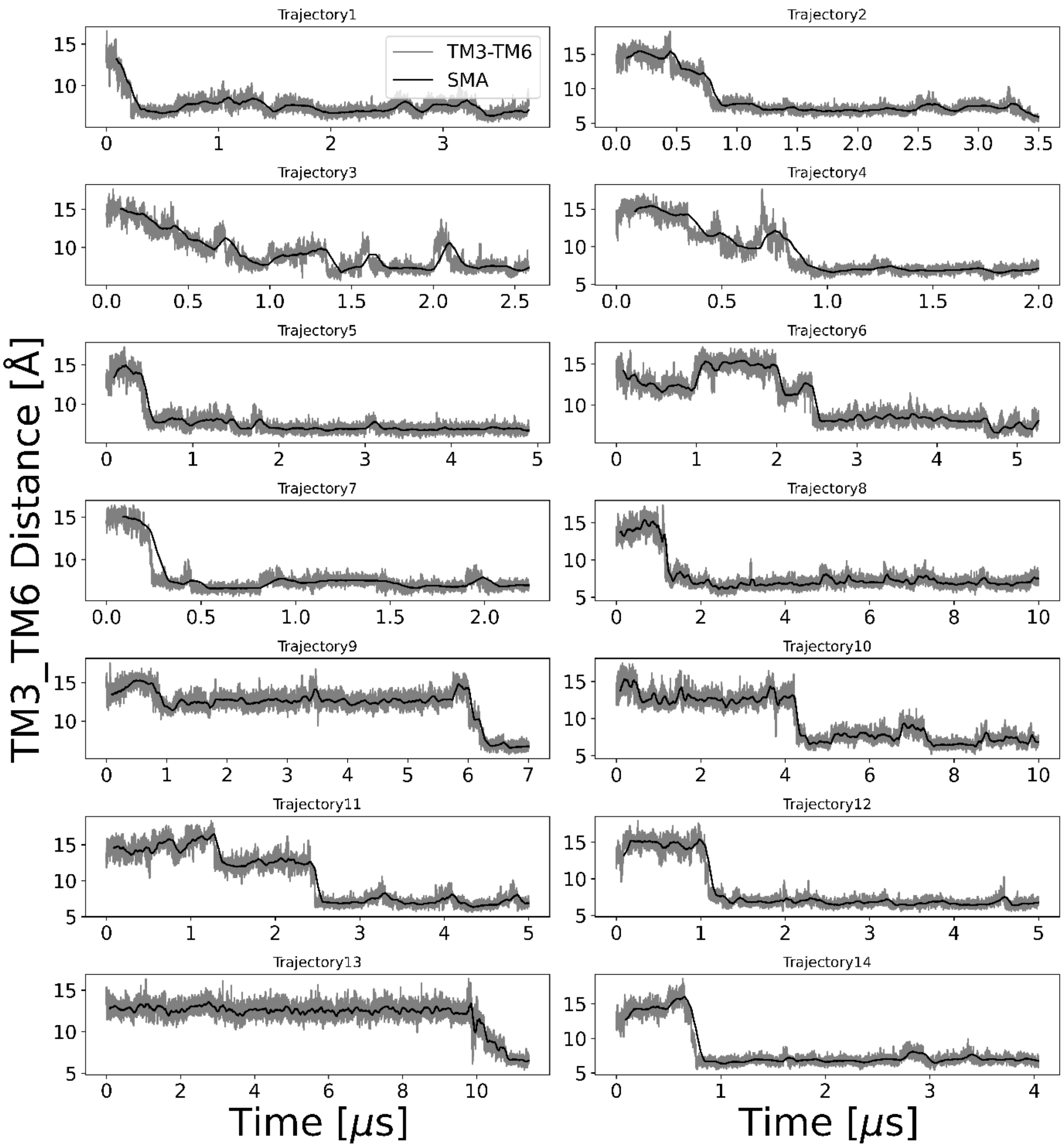

Supplementary Fig. 4. Interhelical TM3-TM6 distances for all β2AR trajectories in the study. All trajectories transition from active to inactive as indicated by the drop in TM3-TM6 distance. A simple moving average (black) is also shown in each plot.

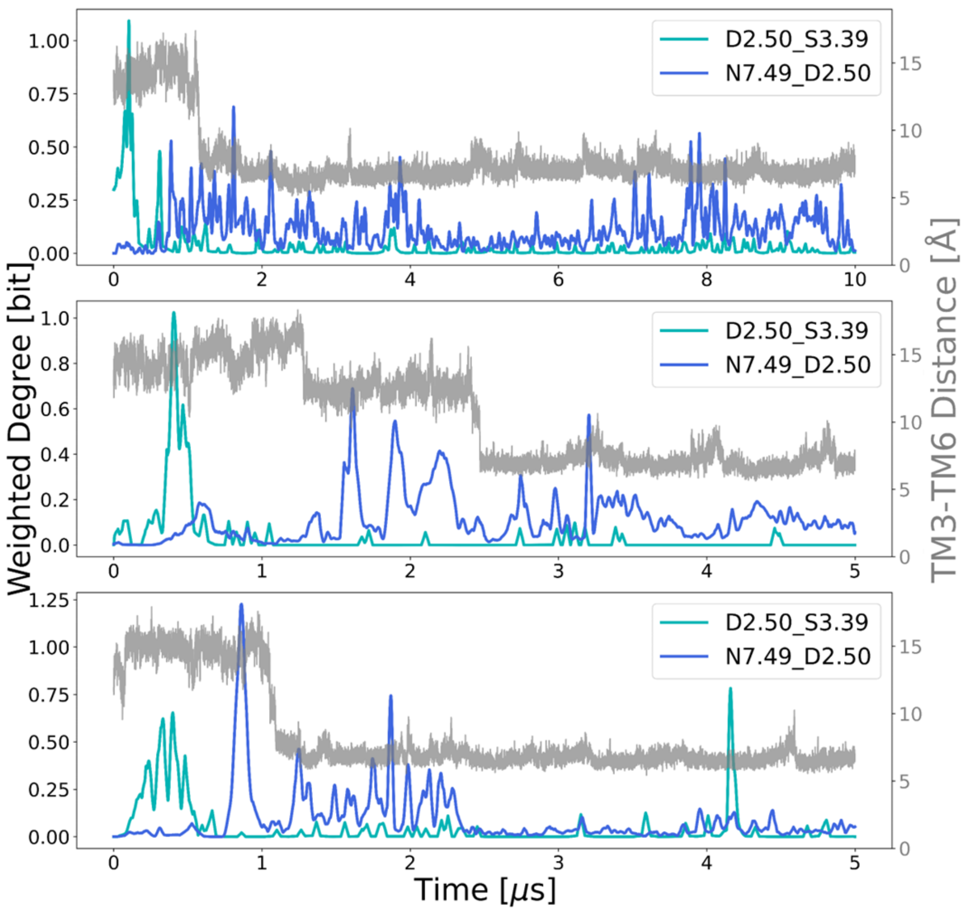

Supplementary Fig. 5. Time-resolved weighted degree plots for D2.50_S3.39 and N7.49_D2.50 contacts from β$_2$AR DRUMBEAT results. Similar to what was found for D$_2$R, these contacts representing a hydrogen bonded core undergo breaking up before deactivation. Notably, the order by which these break is the same as in D$_2$R, D2.50_S3.39 breaks first followed by N7.49_D2.50

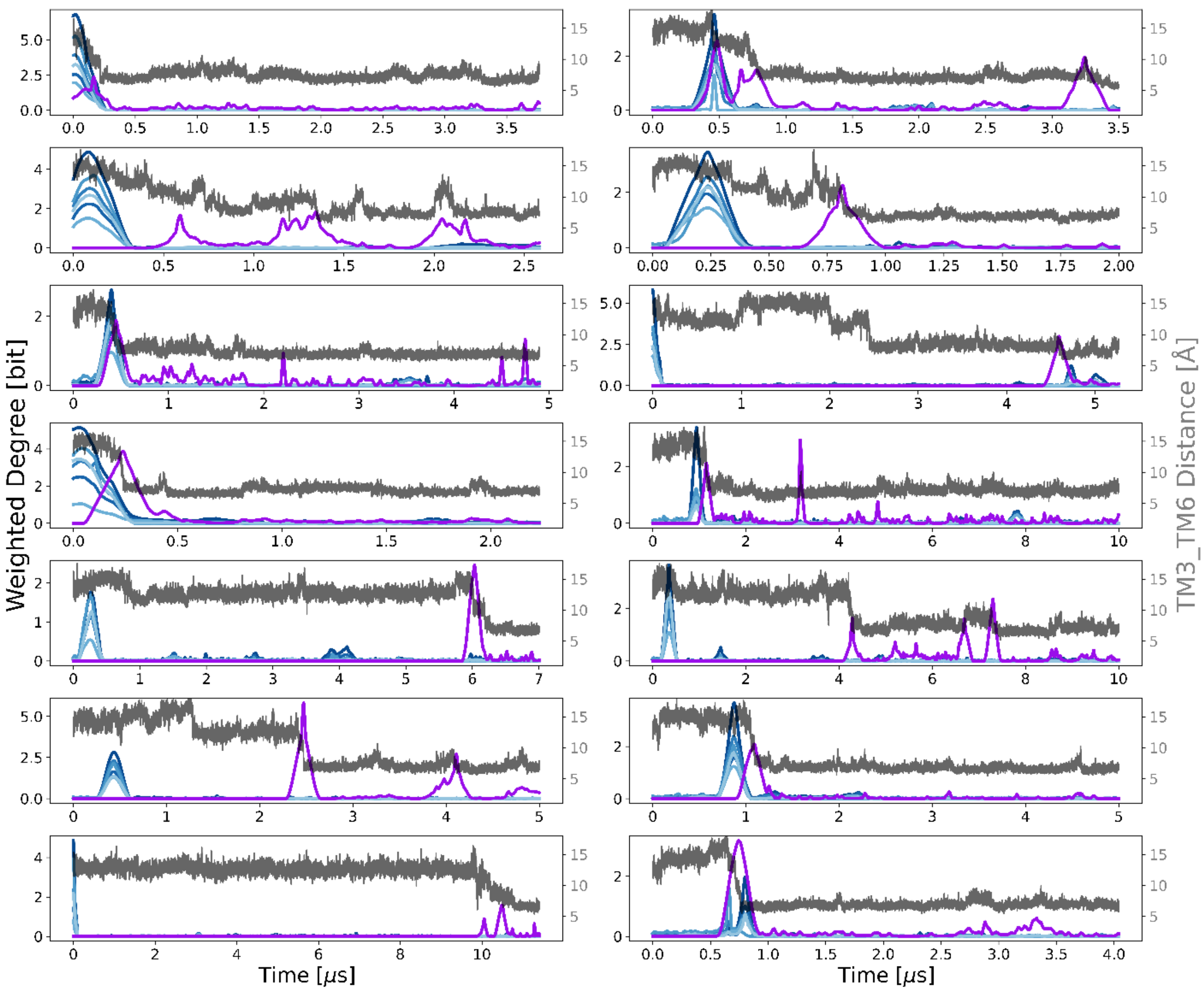

Supplementary Fig. 6. Weighted degree plots for all β2AR trajectories. All plots show TM3-TM6 distance in grey. The weighted degree for enabler and effector TRACs are also shown for each trajectory.

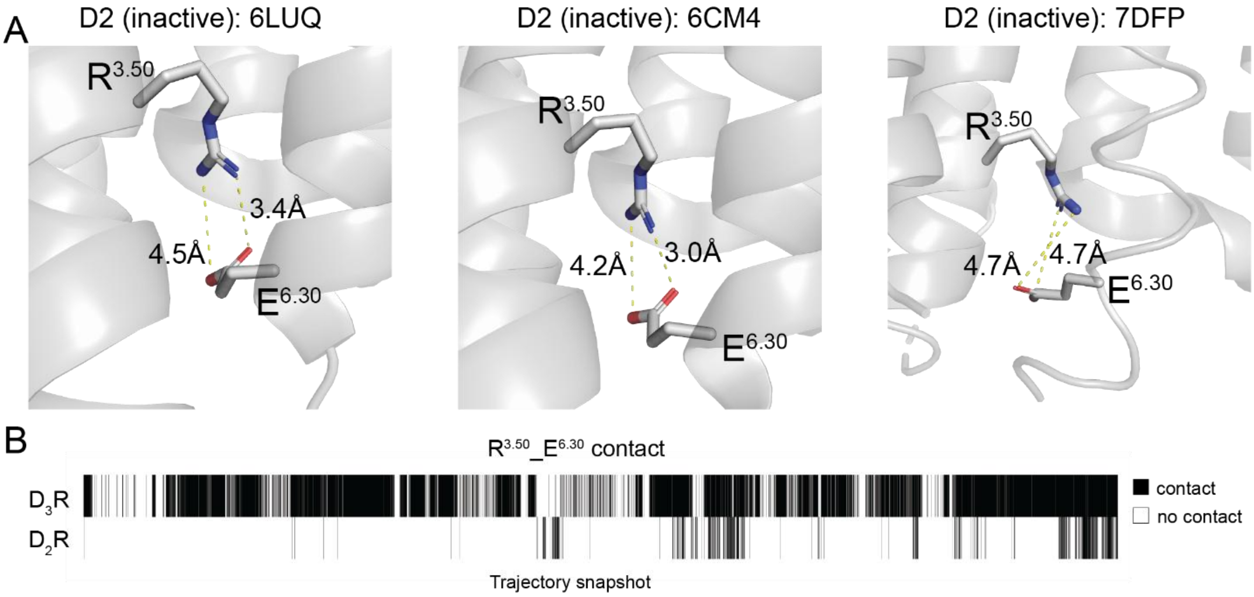

Supplementary Fig. 7. **Structure and contact data for D_2R simulations.** A. Structural representation of three available crystal structures of D_2R demonstrating the minimal distances between R*3.50* and E*6.30* residues. B. Heatmap of R*3.50*_E*6.30* contact appearance throughout D_2R and D_3R inactive state ensemble trajectories. Black color indicates formation of contact in the trajectory snapshot, white – absence.

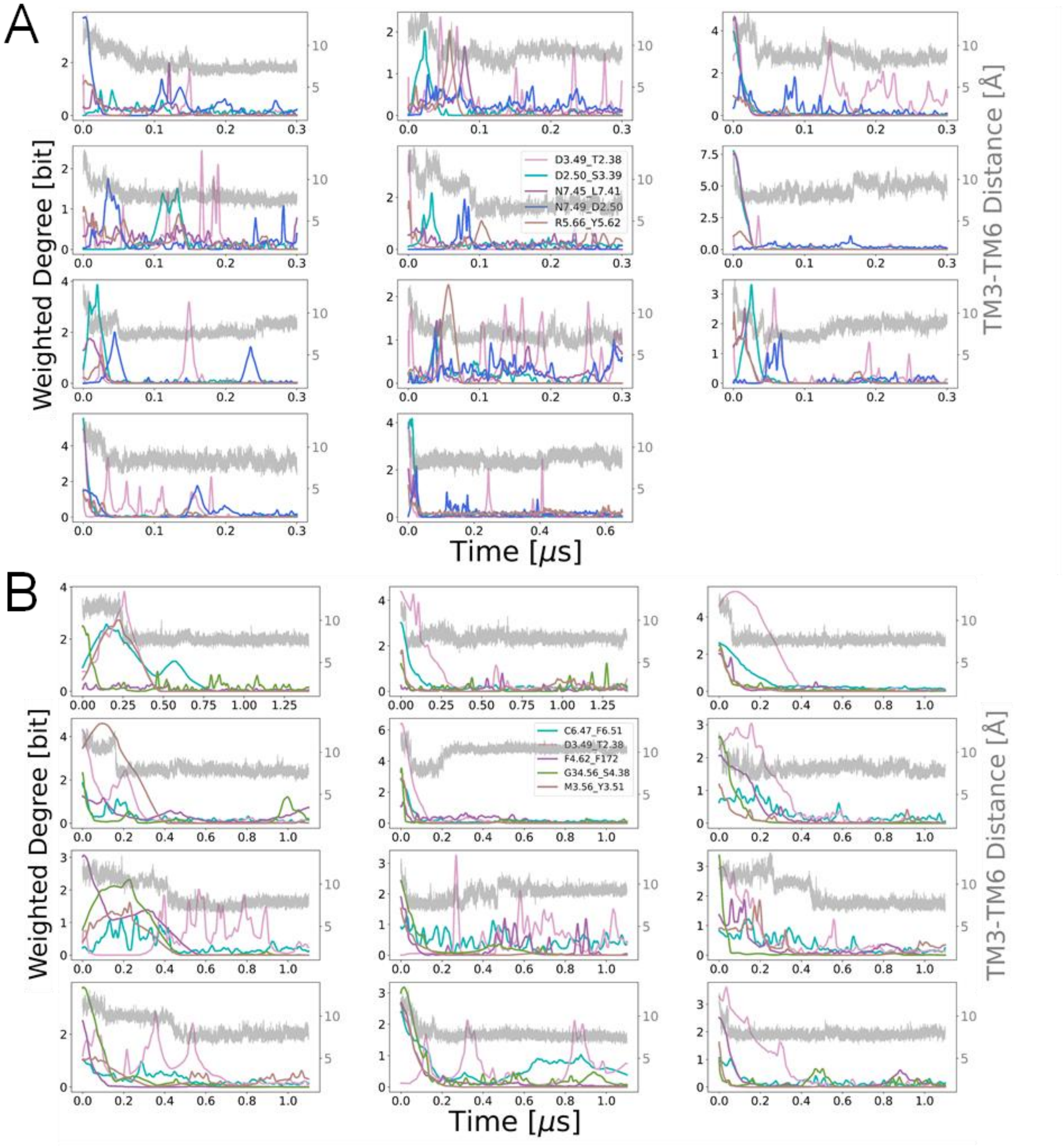

Supplementary Fig. 8. Weighted degree plots for Dopamine results. The plots are shown for all (A) D_2R trajectories and (B) D_3R trajectories. Top five ranked nodes are plotted for each system.

Supplementary Table 1. Swapping mutation between D2R and D3R.

**TRAC**	**Mutation in D_3R**	**Mutation in D_2R**
F4.62_F172 (D_3R)	F172L	L174F
G34.56_S4.38 (D_3R)	G141 deletion	G144 insertion
R5.66_Y5.62 (D_2R)	K216R	R217K

Supplementary Table 2. Summary of computed statistics for swapping mutation study. Two-sided Mann-Whitney U test was performed to establish significant difference between sets.

Comparison (vs groups)		Mann-Whitney Test	
		U statistics	p-value
D_2R	$D_{3/2}R$	30.5	0.0114
D_2R	$D_{2/3}R$	2	0
D_2R	D_3R	9	0.0005
$D_{3/2}R$	$D_{2/3}R$	103	0.2793
$D_{3/2}R$	D_3R	41	0.0283
$D_{2/3}R$	D_3R	65.5	0.0498

Supplementary Table 3. Summary of TRACS determined by DRUMBEAT method for each receptor. A brief description of the functional relevance for each TRAC is also provided. Each TRAC is determined to be either a known microswitch or not, highlighting DRUMBEAT's ability to recapitulate known microswitches while also providing insight into new contacts that facilitate transition.

Protein	DRUMBEAT TRAC residue contacts	Receptor Function Relevance	Known Microswitch
β2AR	R3.50_E6.30	Ionic lock; stabilizes inactive state in many class A GPCRs.[1]	Yes
	C7.54_F8.50	Part of NPxxY/F motif; essential for G-protein coupling.[2]	Yes
	C7.54_V1.53	TM1–TM7 contact; aids structural stability during transitions.	No
	R8.51_C7.54	Supports helix 8-TM7 interface; involved in signaling specificity.	No
	F8.50_Y7.53	Aromatic cluster; key in conformational switch (NPxxY region).[3]	Yes
	S8.47_Y7.53	Modifies NPxxY switch dynamics; stabilizes helix 8 rearrangement.	No
	L3.43_Y7.53	Facilitates TM3-TM7 cross-talk during receptor transitions.	No
D_2R	D2.50_S3.39	Core sodium pocket; allosteric regulation in class A GPCRs.[4]	Yes
	N7.49_D2.50	Links NPxxY and sodium pocket; disrupted upon deactivation.[4]	Yes
	N7.45_L7.41	TM7 stability; may support NPxxY function (not a classic switch).	No
	D3.49_T2.38	DRY motif–TM2 link; stabilizes inactive state (subtype specific).	No
	R5.66_Y5.62	TM5 interaction; may aid in cytoplasmic region stability.	No
D_3R	D3.49_T2.38	DRY motif–TM2 link; stabilizes inactive state (subtype specific).	No
	C6.47_F6.51	TM6 toggle switch region; central in activation.[5]	Yes
	M3.56_Y3.51	TM3 contacts; support DRY motif packing and transitions.	No
	F4.62_F172	TM4/loop aromatic contact; may influence subtype signaling.	No
	G34.56_S4.38	TM4 local contact; role not classically recognized.	No

Supplementary Table 4. Inter-residue distance changes in the active and inactive state structures of the TRAC residues for β_2AR, D_2R and D_3R

TRACs in D_2R (GPCRdb residue numbering)	TRAC in D_2R	Smallest distance (Å) in active structure 8IRS	Shortest distance (Å) in inactive structure 6LUQ
N7.45_L7.41	N418_L414	3.7	6.8
N7.49_D2.50<br>(a part of extended sodium binding site)	N422_D80	2.9	9.1
D2.50_S3.39	D80_S121	3.3	3.5
R5.66_Y5.62	R217_Y213	3.2	2.9
D3.49_T2.38	D131_T68	3.3	2.6

TRACs in D_3R (GPCRdb residue numbering)	TRAC in D_3R	Shortest distance (Å) in active structure 8IRT	Shortest distance (Å) in inactive structure 3PBL
C6.47_F6.51	C341_F345	2.8	4.8
M3.56_Y3.51	M134_Y129	4.2	3.9
F4.62_F172(ECL2)	F170_F172	2.9	3.6
G34.56_S4.38	G141_S146	4.0	3.2
D3.49_T2.38	D127_T63	4.5	3.2

References

1. Latorraca, N. R., Venkatakrishnan, A. J. & Dror, R. O. GPCR Dynamics: Structures in Motion. *Chem Rev* **117**, 139–155 (2017).
2. Fritze, O. *et al.* Role of the conserved NPxxY(x)5,6F motif in the rhodopsin ground state and during activation. *Proc Natl Acad Sci U S A* **100**, (2003).
3. Zhou, Q. *et al.* Common activation mechanism of class A GPCRs. *Elife* **8**, (2019).
4. Katritch, V. *et al.* Allosteric sodium in class A GPCR signaling. *Trends Biochem Sci* **39**, 233–244 (2014).
5. Shi, L. *et al.* β2 Adrenergic Receptor Activation: Modulation of the proline kink in transmembrane 6 by a rotamer toggle switch. *Journal of Biological Chemistry* **277**, 40989–40996 (2002).
