# Supplementary material for: Temporally resolved and interpretable machine learning model of GPCR conformational transition
Source: Nat Commun. 2025 Dec 6;17:257. doi: 10.1038/s41467-025-66958-4 (PMC12783649; doi:10.1038/s41467-025-66958-4)
Supplement: Supplementary file 2 — Description of Additional Supplementary Files [file 41467_2025_66958_MOESM2_ESM.pdf]

Description of Additional Supplementary Files

File name: Supplementary Data 1
Description: Graph source files in .dot format of the universal Bayesian networks generated in this study. The dataset includes: β2AR universal graph, D2R universal graph, D3R universal graph.
